# Supplementary material for: Human innate lymphoid cell activation by adenoviruses is modified by host defense proteins and neutralizing antibodies
Source: Front Immunol. 2022 Oct 5;13:975910. doi: 10.3389/fimmu.2022.975910 (PMC9579290; doi:10.3389/fimmu.2022.975910)
Supplement: Supplementary Table 1 — Summary of candidate receptors based on scRNAseq analyses of human ILCs. ScRNAseq data of candidate receptors by ILCs extracted from the results matrix published by Ercolano et al. Raw sequencing data were normalized to 1 million transcripts (nTPM). [file DataSheet_1.pdf]

Supplementary material

Table S1

	D#1			D#2			D#3		
	ILC1	ILC2	ILC3	ILC1	ILC2	ILC3	ILC1	ILC2	ILC3
**CAR**	0,1	3,5	0	0,5	5	0,3	2,1	2,4	0
**HLA-ABC**	2866	2287	2534	4233	2954	3516	2641	2921	2558
**CD46**	285	340	391	205	201	224	200	242	277
**CD49d**	219	167	265	135	111	206	256	93	253
**DSG2**	2	42	10	0	16	0	1	24	5
**CD69**	1216	3266	2109	1869	1453	1789	1950	2355	2111
**CD161**	200	920	599	146	128	521	147	984	580
**CD80**	0,6	0	0	0,8	0	0,6	1,3	0	0
**CD86**	0	2	7	1	68	49	4	3	14
**HLA-DR**	0,1	0	0	0	0	0	0	0	0
**CD16**	0	1,8	2,8	0	0,3	0,7	0,1	0	0
**TLR2**	2	10	26	1	32	45	5	10	19
**TLR3**	0,3	0,1	0	1,0	0	0,6	3,5	2,0	7,4
**TLR4**	1,8	7,4	0	0,7	67	21	0,9	8	14
**TLR9**	0,3	0,1	0,1	0,1	0	0	0	0	0,3

Figure S1

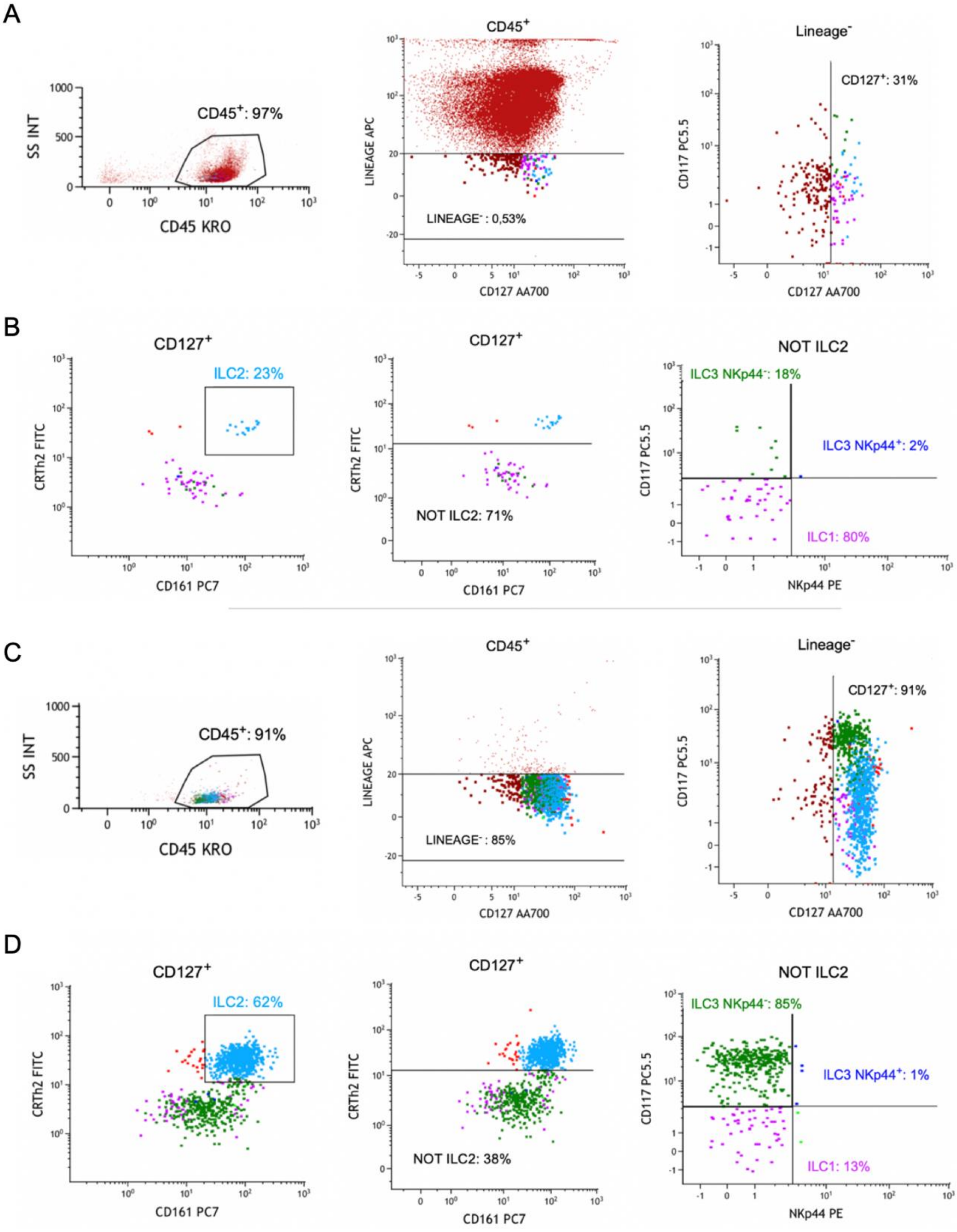
A
CD45+: 97%
SS INT
CD45 KRO
CD45+
LINEAGE APC
LINEAGE- : 0,53%
CD127 AA700
Lineage-
CD127+: 31%
CD117 PC5.5
B
CD127+
ILC2: 23%
CRTh2 FITC
CD161 PC7
NOT ILC2: 71%
NOT ILC2
ILC3 NKp44-: 18%
ILC3 NKp44+: 2%
ILC1: 80%
NKp44 PE
C
CD45+: 91%
LINEAGE- : 85%
CD127+: 91%
D
ILC2: 62%
NOT ILC2: 38%
ILC3 NKp44-: 85%
ILC3 NKp44+: 1%
ILC1: 13%

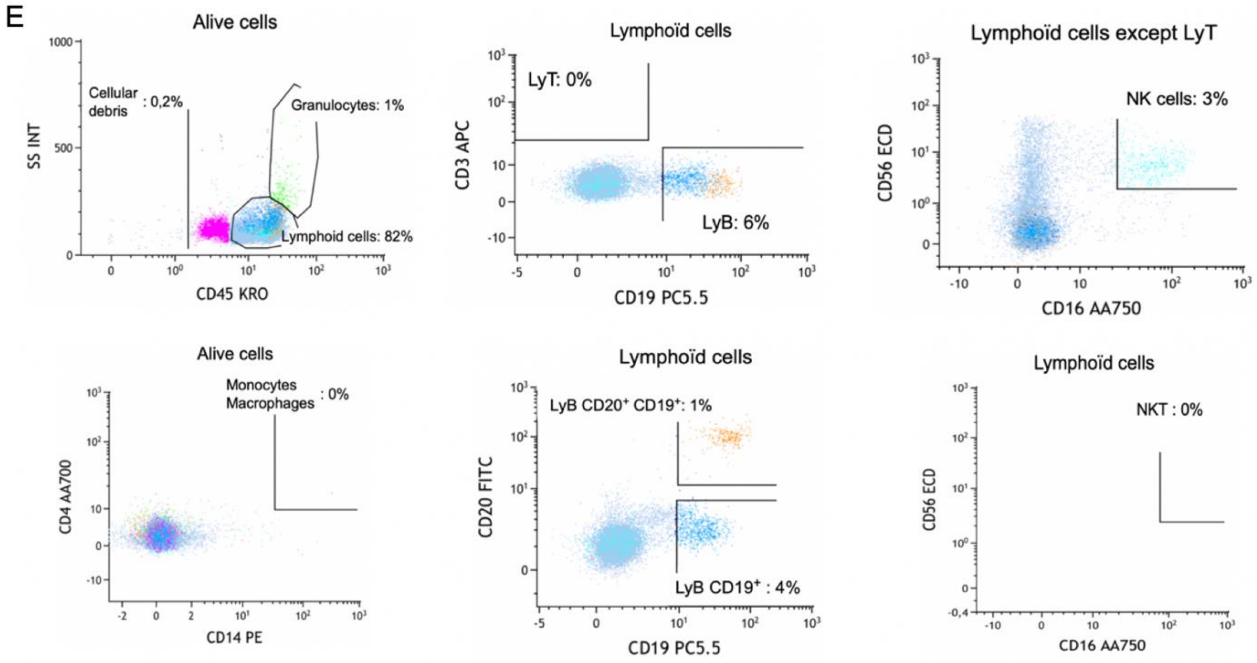

After depletion of cells belonging to a lineage:

- Enrichment of Lin$^-$ CD127$^+$ cells
- 0,4-7% of residual B cells
- 0-2% of T cells
- 0-5% of NKT cells

Optimal medium used:
RPMI + Human Ab serum (10%) + Penicillin-Streptomycin + IL-7 (10 ng/mL) + Pyruvate (1 mM)

Figure S2

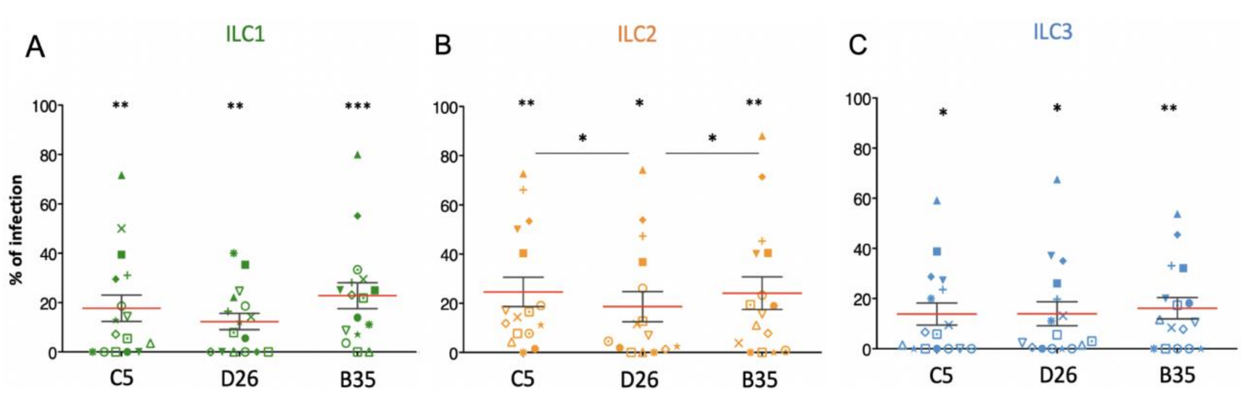

Figure S3

A

Medium / Marker	MFI GFP -	MFI GFP +	Ratio MFI+/MFI-	Fold change	gMFI GFP -	gMFI GFP +	Ratio gMFI+/gMFI-	Fold change
Total ILCs	416	2221	5,34	1	341	2777	8,14	1
HLA-ABC	616	85467	138,75	25,99	565	81889	144,94	17,80
ILC1	426	10	0,023	1	346	10	0,03	1
HLA-ABC	864	91083	105,42	4491	864	76342	88,36	3057
ILC2	312	10	0,032	1	291	10	0,03	1
HLA-ABC	10	76744	7674	239441	10	71407	7140,70	207794
ILC3	462	2179	4,72	1	367	2777	7,57	1
HLA-ABC	10	71350	7135,00	1513	10	70794	7079,40	936

B

Medium / Marker	MFI -	MFI +	Ratio MFI+/MFI-	Fold change	gMFI -	gMFI +	Ratio gMFI+/gMFI-	Fold change
Total ILCs	2047	7140	3,49	1	1557	7456	4,79	1
CD46	3391	71483	21,08	6,04	3635	65995	18,16	3,79
CD49d	3772	43704	11,59	3,32	3546	41930	11,82	2,47
ILC1	2074	7247	3,49	1	1556	7869	5,06	1
CD46	10	70219	7022	2010	10	61601	6160,10	1218
CD49d	10	38263	3826,30	1095	10	35466	3546,60	701
ILC2	1740	6383	3,67	1	1533	6791	4,43	1
CD46	3416	82628	24,19	6,59	3416	75981	22,24	5,02
CD49d	3277	40454	12,34	3,37	3277	41842	12,77	2,88
ILC3	2097	7214	3,44	1	1582	7566	4,78	1
CD46	10	71353	7135,30	2074	10	67961	6796,10	1421
CD49d	4634	44897	9,69	2,82	4620	43780	9,48	1,98

C

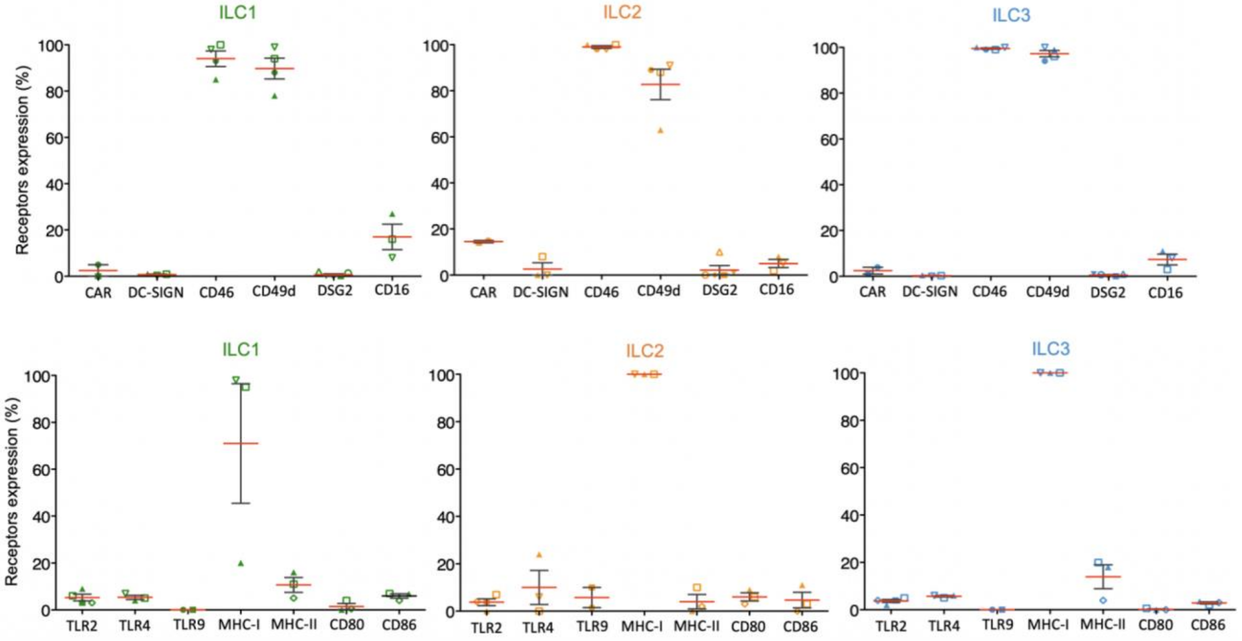

Figure S4

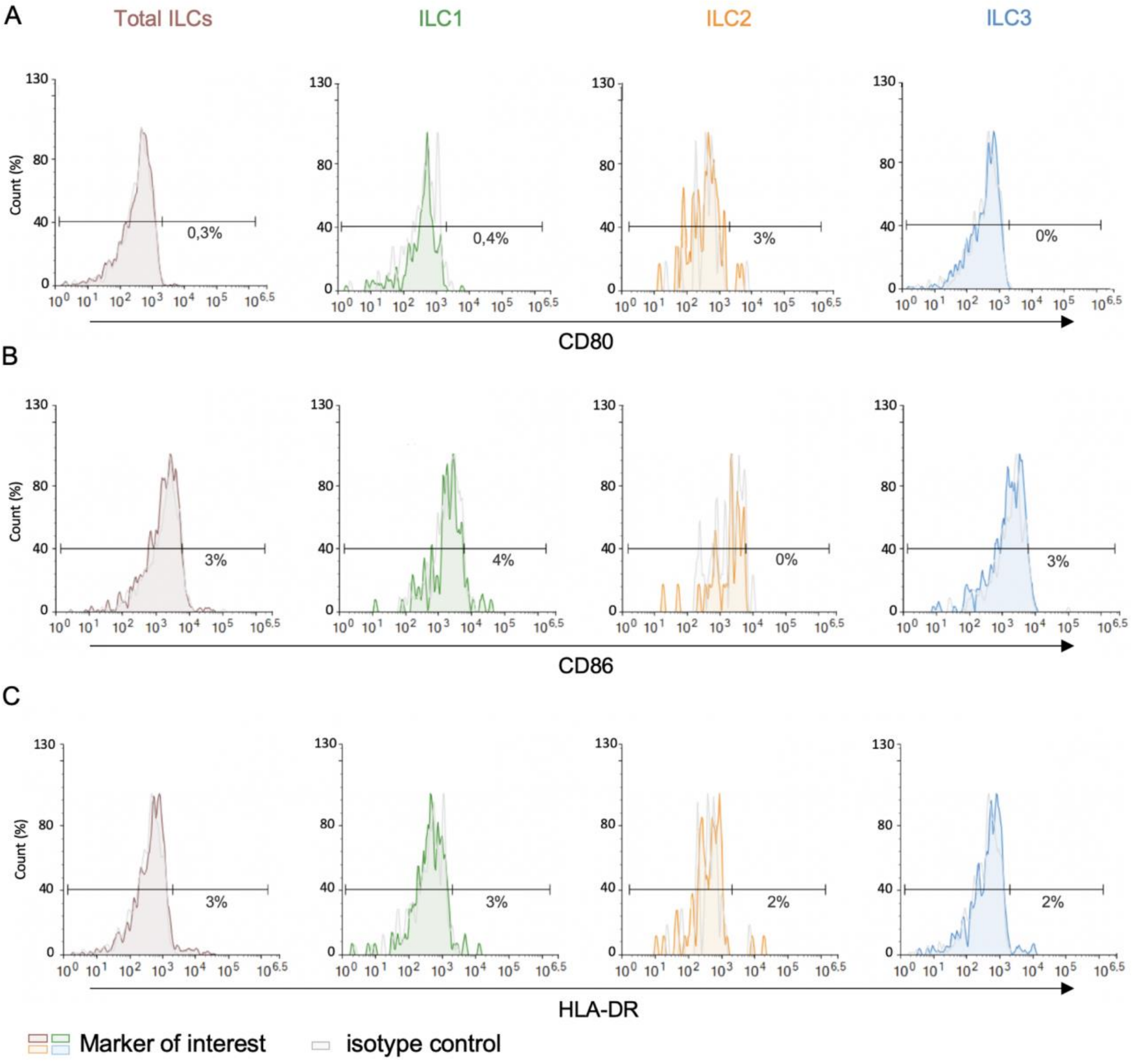

A
Total ILCs
ILC1
ILC2
ILC3
Count (%)
0,3%
0,4%
3%
0%
CD80
B
3%
4%
0%
3%
CD86
C
3%
3%
2%
2%
HLA-DR
Marker of interest
isotype control

Figure S5

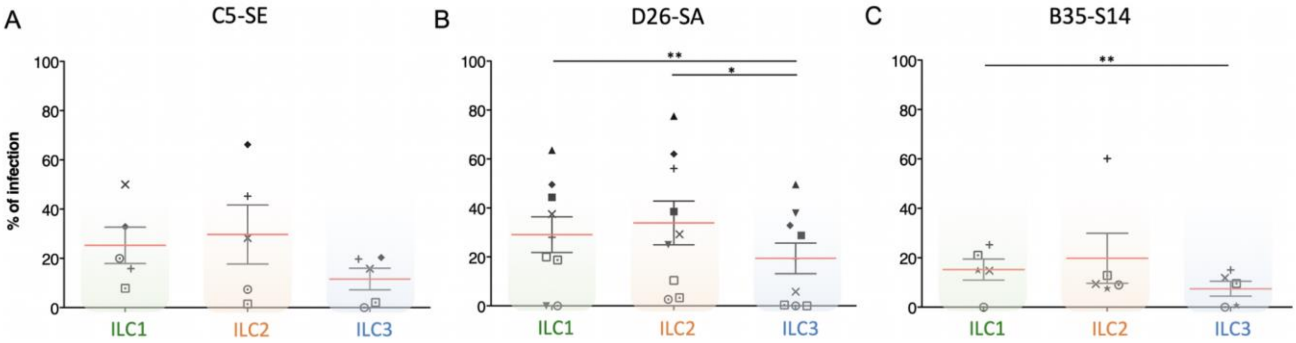

D

Total ILCs / Virus	MFI GFP -	MFI GFP +	Ratio MFI+/MFI-	Fold change	gMFI GFP -	gMFI GFP +	Ratio gMFI+/gMFI-	Fold change
HAdV-C5	879	2592,5	2,95	1	695,5	2715	3,90	1
HAdV-C5-SE	881	2550	2,89	0,98	702,5	2634,5	3,75	0,96
HAdV-D26	953	2467	2,59	1	734	2581	3,52	1
HAdV-D26-SA	823,5	2885	3,50	1,35	623,5	2986	4,79	1,36
HAdV-B35	879	2749,5	3,13	1	719	3661,5	5,09	1
HAdV-B35-S14	915,5	2629,5	2,87	0,92	695	2995	4,31	0,85

ILC1 / Virus	MFI GFP -	MFI GFP +	Ratio MFI+/MFI-	Fold change	gMFI GFP -	gMFI GFP +	Ratio gMFI+/gMFI-	Fold change
HAdV-C5	923,5	2689,5	2,91	1,00	705,5	2749,5	3,90	1,00
HAdV-C5-SE	1075,5	2647	2,46	0,85	602,5	2701,5	4,48	1,15
HAdV-D26	723	2666,5	3,69	1	686,5	2764	4,03	1
HAdV-D26-SA	862	2923,5	3,39	0,92	645,5	2918,5	4,52	1,12
HAdV-B35	945,5	2370,5	2,51	1	988,5	2806,5	2,84	1
HAdV-B35-S14	894	2559	2,86	1,14	821	2647	3,22	1,14

ILC2 / Virus	MFI GFP -	MFI GFP +	Ratio MFI+/MFI-	Fold change	gMFI GFP -	gMFI GFP +	Ratio gMFI+/gMFI-	Fold change
HAdV-C5	1118,5	2789,5	2,49	1,00	938,5	2865	3,05	1,00
HAdV-C5-SE	983,5	2946	3,00	1,20	852,5	2887	3,39	1,11
HAdV-D26	1143,5	2556,5	2,24	1	798,5	2641,5	3,31	1
HAdV-D26-SA	1007	4783,5	4,75	2,12	910	4680	5,14	1,55
HAdV-B35	946	2782,5	2,94	1	829,5	3363	4,05	1
HAdV-B35-S14	1036,5	2660,5	2,57	0,87	887,5	2937,5	3,31	0,82

ILC3 / Virus	MFI GFP -	MFI GFP +	Ratio MFI+/MFI-	Fold change	gMFI GFP -	gMFI GFP +	Ratio gMFI+/gMFI-	Fold change
HAdV-C5	915,5	2525,5	2,76	1	719,5	2618	3,64	1
HAdV-C5-SE	940,5	2707,5	2,88	1,04	756,5	2716	3,59	0,99
HAdV-D26	1023	2392	2,34	1	846,5	2528,5	2,99	1
HAdV-D26-SA	883,5	2462	2,79	1,19	682	2505	3,67	1,23
HAdV-B35	1033,5	3948,5	3,82	1	809,5	4784	5,91	1
HAdV-B35-S14	928	2722	2,93	0,77	695,5	3302	4,75	0,80

Figure S6

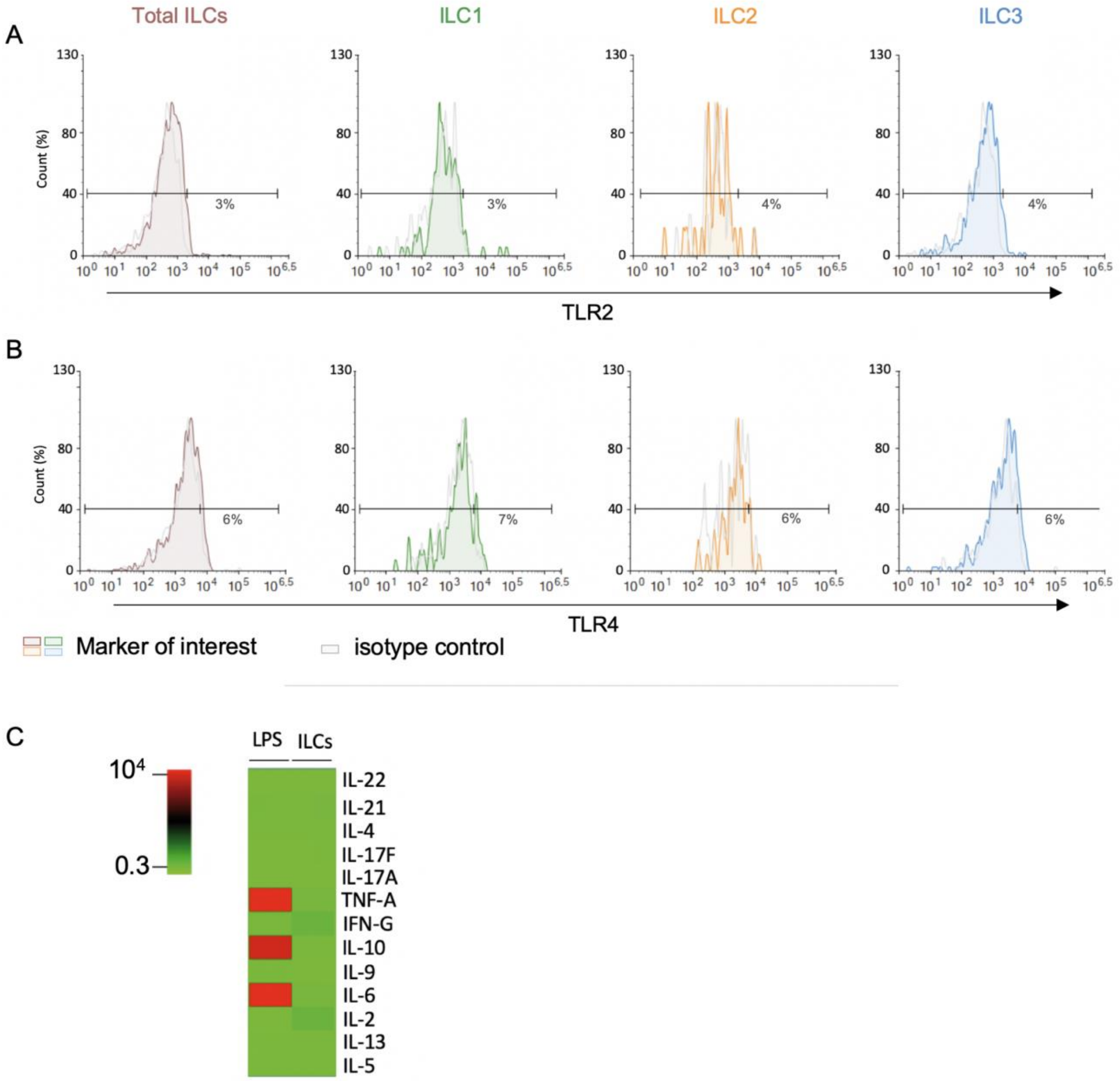
A
Total ILCs
ILC1
ILC2
ILC3
Count (%)
3%
3%
4%
4%
TLR2
B
6%
7%
6%
6%
TLR4
Marker of interest
isotype control
C
LPS
ILCs
IL-22
IL-21
IL-4
IL-17F
IL-17A
TNF-A
IFN-G
IL-10
IL-9
IL-6
IL-2
IL-13
IL-5

Figure S7

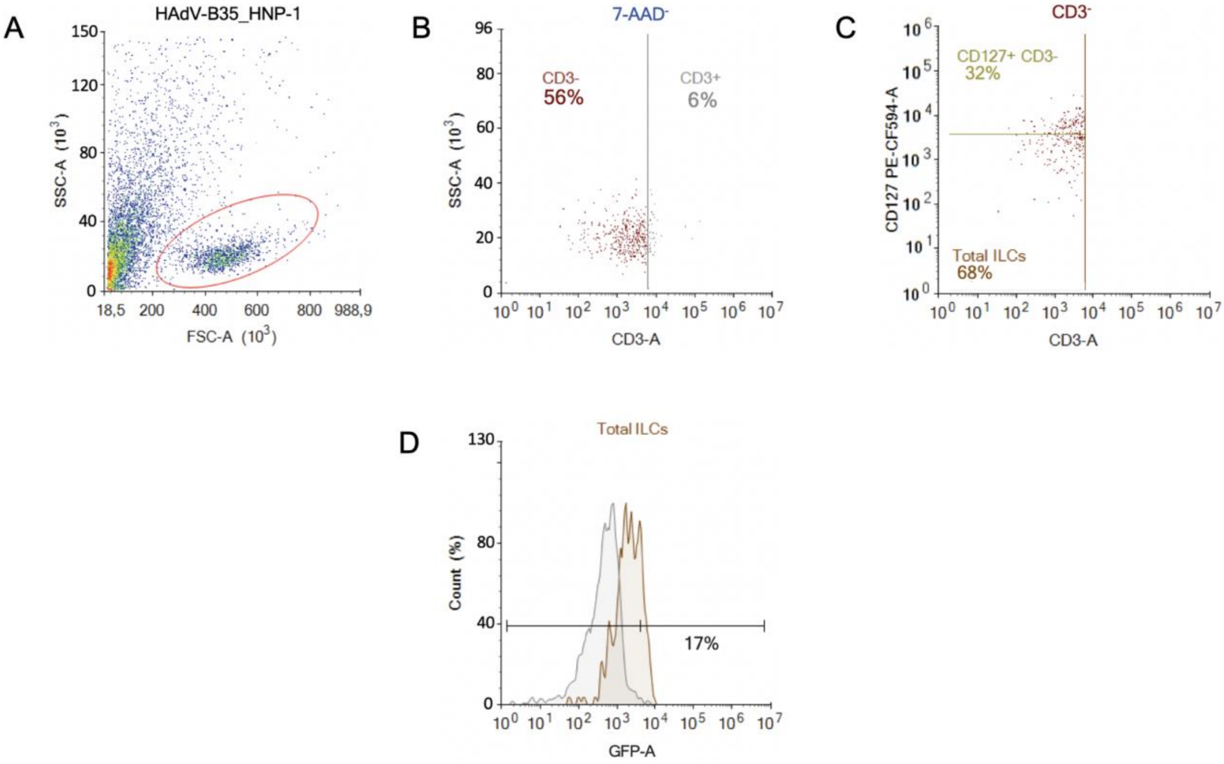

Figure S8

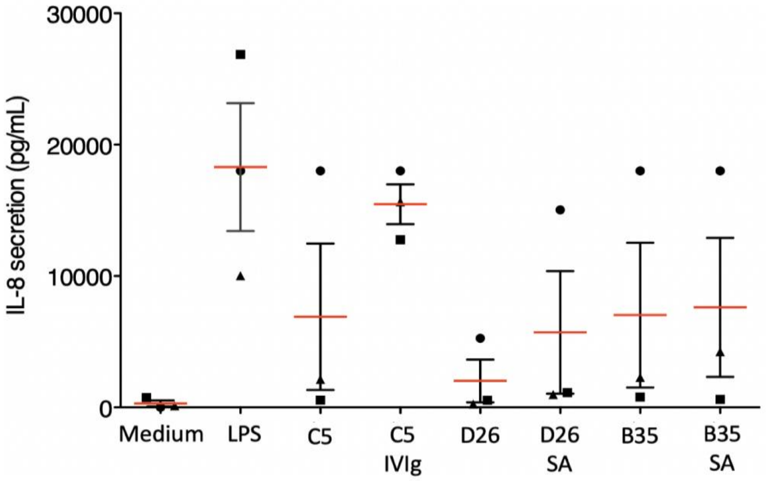

Figure S9

A.

GFP	HAdV-C5		HAdV-D26		HAdV-B35	
	Virus	IC	Virus	IC	Virus	IC
ILC1	+ +	↗	+	↗	+ + +	↘
ILC2	+ + +	↗	+ +	↗	+ + +	↘
ILC3	+	↘	+	↗	+ +	↘

B.

CD69	HAdV-C5		HAdV-26		HAdV-B35	
	Virus vs medium	IC vs virus	Virus vs medium	IC vs virus	Virus vs medium	IC vs virus
ILC1	=	=	+	↘	-	=
ILC2	+	↘	+ +	↘	=	↘
ILC3	-	↘	-	↘	=	↘

C.

Cytokines	HAdV-C5		HAdV-D26		HAdV-B35	
	Virus vs medium	IC vs virus	Virus vs medium	IC vs virus	Virus vs medium	IC vs virus
IL-1β		+	+	-	+	+ +
TNF	+/-	+	+/-	+	+ +	+
IFN-λ_1	+/-	-	+/-	-	+/-	n.d
IFN-$\lambda_{2/3}$	+	+ +	+ +	+ +	+ + +	n.d
IL-8	+	+		+		n.d
IFN-β		+	+	-	+	n.d
IFN-γ	+/-	+	+	-	+	n.d
IL-6	+/-	+	+/-	+	+/-	+
IL-21	+/-	+		+	+	=
IL-5		+				
IL-9					+	n.d

D.

Mo-DCs	HAdV-C5		HAdV-D26		HAdV-B35	
	Virus vs medium	IC vs virus	Virus vs medium	IC vs virus	Virus vs medium	IC vs virus
GFP	+	↗	+	↗	+ +	↗
CD86	+	↗	+ +	↗	+	↘

E.

CD69	HAdV-C5		HAdV-D26		HAdV-B35	
	Virus vs medium	IC vs virus	Virus vs medium	IC vs virus	Virus vs medium	IC vs virus
ILC1	-	↗	+	=	+	↗
ILC2	-	↗	+	↘	-	↗
ILC3	+	↗	+	↘	+	↗

F.

Cytokines	HAdV-C5		HAdV-D26		HAdV-B35	
	Virus vs medium	IC vs virus	Virus vs medium	IC vs virus	Virus vs medium	IC vs virus
IL-8	+	+ +	+	+ +	+ +	+
CXCL10	+ +	-	+ +	-	+ +	-
IL-6	+ +	+ +	+	+ +	+ +	n.d
TNF	+	+ + +	+	+ +	+	+ +
IFN-λ_1	+	+	+ +	+ + +	+	=

Legends

+ = increase compared to the control / - = decrease compared to the control / « = » = equal to the control

↘ *= decrease compared to HAdV alone /* ↗ *= increase compared to HAdV alone*

(grey) No variation compared to cells alone

n.d : not determined
